# Study of the antimicrobial activity of zinc oxide nanostructures mediated by two morphological structures of leaf extracts of *Eucalyptus radiata*

**DOI:** 10.1016/j.heliyon.2024.e25590

**Published:** 2024-02-07

**Authors:** Eric Kwabena Droepenu, Eric Amenyogbe, Mercy Adusei Boatemaa, Evelyn Opoku

**Affiliations:** a Department of Water Resources and Aquaculture Management, School of Sustainable Development, University of Environment and Sustainable Development, Somanya, Eastern Region, Ghana; b Department of Biomedical Engineering, School of Biomedical and Allied Health Sciences, All Nations University, Koforidua, Eastern Region, Ghana

**Keywords:** *Eucalyptus robusta sm*, Nanostructures, Calcination temperature, Antimicrobial, Antifungal, Spectroscopy

## Abstract

The growing microbial resistance against antibiotics and the development of resistant strains has shifted the interests of many scientists to focus on metallic nanoparticle applications. Although several metal oxide nanoparticles have been synthesized using green route approach to measure their antimicrobial activity, there has been little or no literature on the use of *Eucalyptus robusta Smith* aqueous leaf extract mediated zinc oxide nanoparticles (ZnONPs). The study therefore examined the effect of two morphological nanostructures of *Eucalyptus robusta Sm* mediated ZnONPs and their antimicrobial and antifungal potential on some selected pathogens using disc diffusion method. The samples were characterized using Scanning and Transmission Electron Microscopy, Energy-Dispersive Spectroscopy and Fourier Transform Infrared Spectroscopy. From the results, the two ZnO samples were agglomerated with zinc oxide nanocrystalline structure sample calcined at 400 °C (ZnO NS_400_) been spherical in shape while zinc oxide nanocrystalline structure sample calcined at 60 °C (ZnO NS_60_) was rod-like. The sample calcined at higher temperature recorded the smallest particle size of 49.16 ± 1.6 nm as compared to the low temperature calcined sample of 51.04 ± 17.5 nm. It is obvious from the results that, ZnO NS_400_ exhibited better antibacterial and antifungal activity than ZnO NS_60_. Out of the different bacterial and fungal strains, ZnO NS_400_ sample showed an enhanced activity against *S. aureus* (17.2 ± 0.1 mm) bacterial strain and *C. albicans* (15.7 ± 0.1 mm) fungal strain at 50 mg/ml. Since this sample showed higher antimicrobial and antifungal activity, it may be explored for its applications in some fields including medicine, agriculture, and aquaculture industry in combating some of the pathogens that has been a worry to the sector. Notwithstanding, the study also provides valuable insights for future studies aiming to explore the antimicrobial potential of other plant extracts mediated zinc oxide nanostructures.

## 1 Introduction

Antimicrobial resistance has been a challenging issue currently to the word health care system. Also, multidrug-resistant diseases have had a major impact on antibacterial therapy. In lieu of this. Researchers are delving into the development of effective antimicrobial agents to combat the increasing threat of some pathogens due to their resistance to some drugs [1]. Notwithstanding, natural products, particularly plant extracts have gained significant attention as potential sources of antimicrobial compounds [2]. Incorporating these plant extracts in the synthesis of metal oxide nanomaterials have contributed immensely in the therapeutic applications of these drugs and thus proved itself a new source of antimicrobials [3].

Nanotechnology is an emerging research field where particle size of a material is reduced to nanometer scale range (<100 nm). This field has shown applications in a range of different fields of science, and especially in the medical sciences [4]. The interest in nanoparticles (NPs) of late is as a result of their useful properties. These include their specific catalytic, magnetic, electronic, optical, antifungal, antimicrobial, wound healing and anti-inflammatory properties [[5], [6], [7], [8]].

Studies have shown that, some inorganic oxide NPs synthesized biologically have shown an enhanced antibacterial activity. For instance, iron nanoparticles (FeNPs) and Barium oxide nanoparticles (BaONPs) which were synthesized using *Enterobacter* bacterial strain G52 biomass and *Spirogyra-hyalina* respectively showed potent antibacterial and antifungal potential against selected microbial strains [9,10]. Notwithstanding, zinc oxide (ZnO) nanoparticles (ZnONPs) synthesized using *Monotheca buxifolia* has also shown a range of biological and environmental applications [11]. These route of synthesis which is referred to as green synthesis have comparatively lower environmental impact as compared to the chemical routes [12,13].

Literature documents the use of oleic acid, gelatin, albumin, starch, and alginate with fungus, algae, bacteria, plant extracts, and other living things to fabricate NPs [14]. While the approach of fabricating NPs from bacteria, algae, and fungus have numerous benefits, it also has some significant challenges, such as bacterial toxicity, capacity to grow germs in isolation, and the incubation process [15]. However, plant extracts are considered safer to some of these organisms. The plant extracts are reported to contain various types of metabolites (proteins, lipids, phenolic compounds, polysaccharides, sugar etc) which have reduced and stabilizing agents such as phenols, flavonoids, tannins, and terpenoids. These bioactive constituents have been reported to aid in the reduction, chelation, stabilization, and production of NPs [16]. These bioactive constituents also play some role in the control of the NPs morphology as reported in literature [6]. Morphology and high specific surface-to-volume ratio of NPs enhance their greater mobility through biological cell membranes [17]. These properties provide higher adsorption sites, hence affect antibacterial activities [18,19]. The morphology of NP plays a crucial role in the determination of their physicochemical and biological activities. It has been observed that, the morphological characteristics of ZnO NPs can be influenced by many factors as reported in literature [20]. In this context, utilizing plant extracts as reducing and stabilizing agents for the synthesis of ZnO NPs offers a greener and more sustainable approach to conventional chemical methods.

Literature has documented a substantial number of studies into medicinal plants used in the synthesis of NPs and their antimicrobial potentials against some selected pathogens. *Mentha arvensis*-mediated magnesium oxide nanomaterial (MgONPs) and *Paraclostridium benzoelyticum* bacterium-mediated ZnONPs showed varied degree of inhibition against *Helicobacter felis*, *Helicobacter suis, Helicobacter salomonis,* and *Helicobacter bizzozeronii* [21,22]. Other plant mediated NPs whose antimicrobial potentials were investigated include *Allium sativum* [23], *Calotropis procera* [24], *Mentha spicata* [25], *Pnica granatum* [26], and pine pollen extracts [27].

Among the vast array of plant species, *Eucalyptus,* originally from Australia is known to be an ethnomedicinal plant. It was used in the treatment of asthma, bronchitis, diarrhoea and respiratory tract infections [28]. The plant belongs to the *Myrtaceae* family with about 900 species and subspecies distributed worldwide [29]. The plant is also known for its aromatic and powerful inhibitory allelopathic impact [30,31]. The plant is well adapted to diverse edaphoclimatic conditions, hence the widespread all over the world. The leaf is noted for its essential oils (EOs) which is used in medicine, perfumery, and the food industry [32]. Various species of this plant has been explored for its antimicrobial potentials. Notable among the species are *Corymbia citriodora, E. dives, E. globulus, E. delegatensis* subsp. *Tasmaniensis, E. pauciflora, E. radiata, E. smithii, E. urophylla*, *E. robusta Sm*, and *E. viminalis* [33,34]. For instance, crude extracts from leaves and EOs of *E. largiflorens, E. intertexta, E. citriodora* L., *E. globulus*, and *E. radiata* were reported to show strong antimicrobial activity against some selected bacteria and fungi [[34], [35], [36], [37], [38]]. However, different NPs were synthesized from these plant species for their multiple biomedical applications. According to Kiran et al. [39], *E. tereticornis* mediated gold NPs (AuNPs) was synthesized and its *in-vitro* antibacterial, antioxidant and anticancer potential was evaluated. The biosynthesized sample gave a positive result for all the tested pathogens. On the other hand, silver NPs were fabricated using *E. camaldulensis* gum and *E. globulus* [[40], [41], [42]] and their antimicrobial potentials were measured against some selected microorganisms. All the synthesized samples exhibited varying inhibition zones on the pathogens. Furthermore, *E. globulus and E. camaldulensis* leaf extract and EOs were also used in the synthesis of zinc oxide and titanium dioxide NPs (ZnONPs and TiO_2_NPs). These plant-mediated NPs were evaluated for their antimicrobial potentials and were reported to manifest excellent results [[42], [43], [44]]. Others also include MgO NPs [45], and Au NPs [39].

Despite the various articles available on the different biosynthesized NPs using different Eucalyptus species, there are limited articles on the use of *E. robusta Smith* in the synthesis of ZnO NPs. *E. robusta Smith* (*Myrtaceae*) is known for its rapid growth. It has a superficial root system, and ovate-lanceolate leaves with a long apex. The leaf measures about 12–20 cm long and 4–8 cm wide. However, Vitta et al. [46], reported that, iron nanoparticles (FeNPs) synthesized from this plant extract showed an inhibitory effect on the selected pathogens based on the particle size of the NPs. In fact, ZnO based materials can initiate antibacterial activity even in the absence of light as reported by Lin et al., [47]. Also, ZnO NPs has proven to have inhibitory effects on the formation of bacterial biofilm [48]. The incorporation of the plant extract into the synthesis process can further enhance the antimicrobial activity of ZnO nanostructures due to the bioactive constituents present in the extract and the eco-friendly nature of the synthesis process.

Given the significant antimicrobial potential of *E. robusta Sm.* leaf extracts and the unique properties of ZnO, this study therefore aims at investigating the effect of two morphological structures of the aqueous leaf extract mediated ZnO nanostructures on their antimicrobial potentials. The objectives of the study are to synthesize nano ZnO samples from the aqueous leaf extracts of *E. robusta Sm.* at different calcination temperatures, characterize the synthesized samples using techniques such as scanning and transmission electron microscopy (SEM, TEM), energy-dispersive spectroscopy (EDX), and infrared spectroscopy (FTIR). Finally, assessing the antimicrobial activities of the synthesized nano ZnO samples against some selected pathogenic microorganisms including *E. coli, S. typhi, S. aureus, K. pneumonia, P. digitatum* and *C. albicans*. The choice of these pathogens are based on their availability at the time of this study. This study therefore has provided valuable insights into the potential of *E. robusta Sm*. leaf extract as a natural source for the synthesis of ZnO nanostructures with enhanced antimicrobial activity.

## 2 Materials & methods

### 2.1 Collection and preparation of plant extracts

Young fresh leaves of *E. robusta Sm.* was harvested from a site in Mampong Akuapem in the Eastern Region of Ghana. The plant species was found to matched with the voucher specimen (Q43) at the National Herbarium of the Royal Botany Gardens, Melborne, Victoria, Australia. The young leaves were adopted for this study because, study shows that, young leaves (*Ilex guayusa Loes* and *Eucalyptus*) are high in bioactive molecules (phenolic compounds, carotenoids, flavonoids, tannic acids, volatile oils [49,50]. The harvested leaves were processed within 32 h to reduce the escape of any volatile compounds [51]. The dried leaf sample was blended into fine powder, stored in air-tight plastic bags in a desiccator at 32 °C.

The current study adopted the method reported by Halanayake et al. [52], with some modifications. A weighed mass of 10 g of the dried powdered leaves of *E. robusta Sm* was mixed with 200 ml of distilled water at a temperature of 60 ± 2 °C for about 25 min. The mixture was filtered using Whatman filter paper and the filtrate was divided into two portions and stored in a clean Schott bottle for further analysis.

### 2.2 Synthesis of plant mediated zinc oxide nanostructures

Two different methods were employed in the synthesis of the nano ZnO samples for this study. The method reported by Perveen et al. [53], was used with some modifications for the sample labelled ZnO NS_60_. Thus, a mixture of 50 mL of 0.2 M Zinc acetate dihydrate (98.0% purity) and 50 mL of aqueous extract of *E. radiata* sample in a 250 ml Schott bottle was given a constant stirring (350 rpm) with a magnetic stirrer on a hot plate at 60 °C for two and a half hours, until a brown precipitate was formed in the solution. The mixture was made basic at a pH of 12 using 2.0 M KOH (90% purity) solution. The solution was centrifuged using Hanil FLETA 5 Centrifuge machine at 4000 rpm for 20 min. The precipitate was further washed with deionized water and then subjected to overnight drying in a hot air oven at a temperature of 60 °C. The dried sample was grinded in a mortar to a fine powder (labelled ZnO NS_60_) and preserved in air tight containers for characterization and antimicrobial studies. In the second sample preparation, the procedure reported by Prathna et al. [54], was adopted with some modifications. Briefly, 30 mL of 0.2 M Zinc acetate dihydrate was mixed with 70 mL of the aqueous extract of *E. radiata* sample and the mixture incubated at 60 °C in a water bath for two and a half hours under shaking conditions (150 rpm) and a pH of 12. This was followed by centrifuging the mixture at 4000 rpm for 20 min, and then washing with deionized water and finally drying in hot air oven at a temperature of 60 °C overnight. The sample (ZnO NS_400_) was calcined at a temperature of 400 °C for 2 h the crystalline sample was then preserved in air tight containers for characterization and antimicrobial studies. Annealing temperature at 400 °C was adopted for this study because, literature has reported that, subjecting the sample to a higher temperature decreases the particle size [55,56]. The synthesis conditions used for the two samples are summarized in Table 1.

**Table 1:** Synthesis condition used for the two synthesized ZnO samples.

Sample	Synthesis condition	Particle size
ZnO NS_60_	Reaction temperature: 60 °C; Reaction time: 2 h; Centrifuging: 4000 rpm for 20 min; Drying temperature: 60 °C; Drying time: 12 h; pH: 12	51.04 ± 17.5
ZnO NS_400_	Reaction temperature: 60 °C; Reaction time: 2 h; Centrifuging: 4000 rpm for 20 min; Drying temperature: 60 °C; Drying time: 12 h; pH: 12; Calcination: 400 °C for 2 h	49.16 ± 1.6

### 2.3 Characterization of plant mediated ZnO nanomaterials

The morphological features of *E. radiata* mediated nano ZnO samples were determined spectroscopically and microscopically. Scanning electron microscopy (SU3500, Hitachi) with an attached spectral imaging system (Thermo Scientific NSS (EDS)) and detector tape (BSE-3D) operating with conditions (10.0 kV voltage; 11.6 mm distance; 40 Pa pressure) was used to determine the elemental composition and purity of the samples. Furthermore, transmission electron microscope (JOEL 1230, Japan) was used in confirming the particle shape and size of the as-prepared nano ZnO samples. Sample preparation followed the procedure reported by Nuraqeelah et al., [57]. Briefly, the nano ZnO sample was placed on an aluminium plate and coated with Platinum for SEM analysis. On the other hand, TEM analysis was carried out by dropping 4 μL of the diluted sample onto a coated copper grid and observed under the microscope. The average particle size of each sample was then determined using IMAGE J software version 1.53t where 10–30 particles were used.

Infrared spectroscopy on the synthesized nano ZnO samples was carried out to determine the surface functional groups with spectrometer (Thermo Scientific Nicolet iS10, USA) at a range of 4000–400 cm^−1^ and resolution of 4 cm^−1^. Potassium bromide powder technique reported by Yang et al. [58], was used for this study.

### 2.4 Entrapment efficiency of ZnO nanostructures (Ns)

The amount of phytochemicals embedded on the fabricated nano ZnO sample was determined using the procedure reported by Sharma et al. [59], with slight modifications. Briefly, 1.0 g each of *E. radiata* mediated nano ZnO samples in 10 ml of ethanol were sonicated for 15 min. The mixture was transferred in clean vials and centrifuged at 4000 rpm for 15 min. The absorbance corresponding to the concentration of the drug was measured using UV–Vis spectrophotometer (UV-1800 Series, SHIMADZU, Japan) at 480 nm. The entrapment efficiency (EF) was calculated using the expression (1):

(1) $$EE=\frac{Total\;amount\;of\;drug\;loaded-Total\;free\;drug\;in\;supernatant}{Total\;amount\;of\;drug\;loaded}\times 100$$

### 2.5 Antimicrobial activity assay

Antimicrobial potential of *E. radiata* mediated nano ZnO samples employed agar well diffusion technique [60] which were tested against two Gram-negative bacteria strains (*E. coli* and *S. typhi*), two Gram-positive bacteria strains (*S. aureus* and *K. pneumoniae*) and two fungal strains; (*C. albicans* and *P. digitatum*). In brief, inoculated plates were allowed to dry in 10 min and a 6 mm equidistant wells were created, and the wells were dispensed with 80 μL of the respective concentrations (50.0, 25.0, 12.5, 6.25 and 3.125 mg/mL) of the nano ZnO samples under aseptic conditions. The plates were dried (30 min) and incubated at 37 °C for 18–24 h and 25 °C for 3–5 days, for bacteria and fungi respectively. The inhibition zone (mm) was measured after the incubation. Three replicates were taken and the mean recorded as ± standard error of the mean (SEM). Ciprofloxacin (15 μg/mL) and fluconazole (150 μg/mL) were used as reference drugs for bacteria and fungi respectively.

## 3 Results and discussions

### 3.1 Morphological analysis

**Fig. 1:**
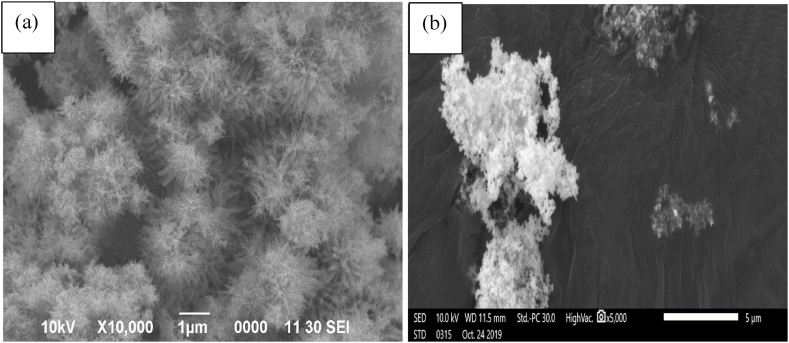
SEM micrograph of *E. radiata* mediated nanostructures for (a) ZnO NS_60_ and (b) ZnO NS_400_.

The surface morphology of the two samples of *E. radiata* mediated nano ZnO samples calcined at two different temperatures are shown in Fig. 1(a and b). The SEM images of the two samples were found to be agglomerated with different morphological structures. Images in Fig. 1 (a) showed rod-like or whiskers-like forms whereas images in Fig. 1 (b) were found to be spherical in nature.

More agglomeration was seen in the sample prepared at a lower calcination temperature (ZnO NS_60_) due to the presence of water molecules in the extracts as compared to the sample prepared at a higher temperature (ZnO NS_400_). Presence of water molecules accounted for the high agglomeration of the as-prepared NPs [61]. Again, Kamarajan et al. [56], also reported that, the higher the calcination temperature, the higher the segregation of the nanoparticles leading to lesser agglomeration.

**Fig. 2:**
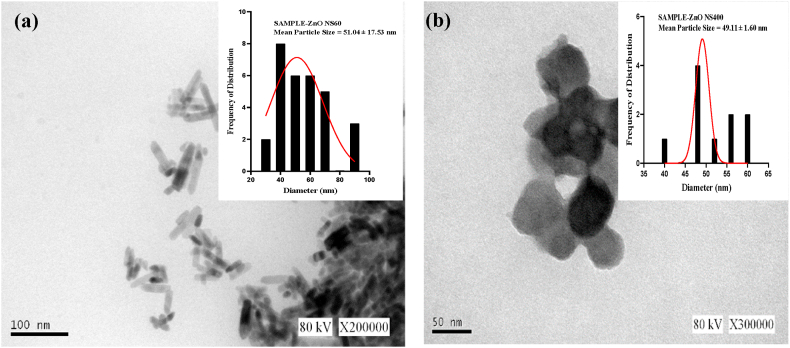
TEM micrograph of *E. radiata* mediated nanostructures for (a) ZnO NS_60_ and (b) ZnO NS_400_.

Fig. 2(a–b) illustrated the TEM images showing rod-like and spherical shaped structures for ZnO NS_60_ and ZnO NS_400_ respectively.

The mean particle size as shown in the histogram distribution (inset) was 51.04 ± 17.5 nm and 49.16. ± 1.6 nm for ZnO NS_60_ and ZnO NS_400_ respectively. Similar structures were synthesized by Kasahun et al. [62], when *Moringa oleifera* leaf extract was used in a green synthesis where thermal decomposition at 400 °C for 3 h was the reaction condition used.

**Fig. 3:**
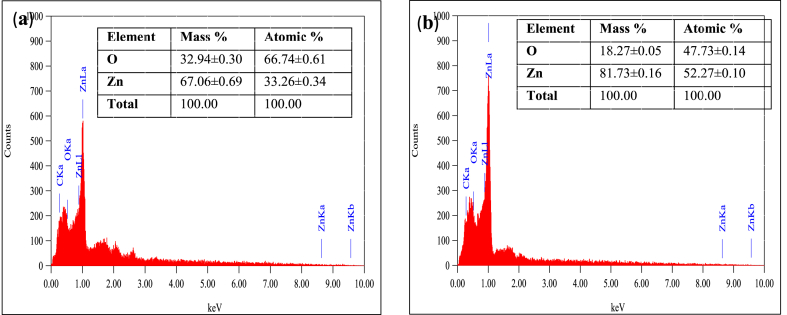
EDS spectra of *E. radiata* mediated nanostructures for (a) ZnO NS_60_ and (b) ZnO NS_400_.

The spectral diagram from the EDX analysis of the two synthesized nano ZnO samples at different calcined temperatures (ZnO NS_60_ and ZnO NS_400_) are illustrated in Fig. 3(a–b). From the spectrum, elemental zinc and oxygen were confirmed as the two major peaks for the two samples. It can be concluded that, as the calcination temperature increased, the percentage average mass of the zinc also increased from 67.06% to 81.73%. These strong signals in the zinc region confirm the formation of ZnO nanoparticles according to Dobrucka & Dugaszewska, [63]. Moreover, the displayed absorption peaks of the samples were due to the surface plasmon resonance of nanocrystalline ZnO nanoparticles [64].

Literature supports this assertion as Kamarajan et al. [56], reported of an increase in the percentage mass of elemental zinc from 72.1% to 78.2% when the calcination temperature of a plant mediated ZnO NPs was increased from 300 °C to 400 °C respectively. The authors also reported that, the presence of oxygen could be generated from the plant phytochemicals (proteins, sugar, and vitamins) which served as reducing agents in stabilizing the nano ZnO sample [65].

### 3.2 Surface functional groups (FT-IR) analysis

**Fig. 4:**
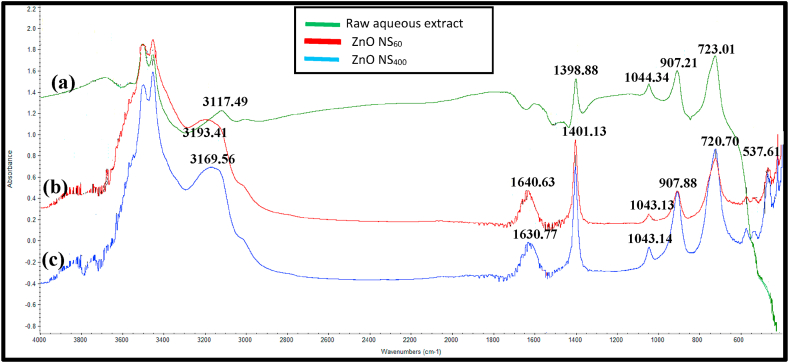
FTIR spectra of (a) raw aqueous extract (b) ZnO NS_60_ and (c) ZnO NS_400_.

The probable reducing and stabilizing biomolecules in the synthesized nano ZnO samples were investigated using infrared spectroscopy. Fig. 4 illustrates the FTIR spectra for (a) *E. radiata* raw aqueous extract, (b) *E. radiata* nano ZnO sample calcined at 60 °C (ZnO NS_60_) and (c) at 400 °C (ZnO NS_400_).

The spectrum of the raw aqueous leaf extract of *E. radiata* exhibited several significant peaks from 3100 to 3600 cm^−1^; and also at 1398.88, and 1044.34 cm^−1^. The broad band between 3100 and 3600 could be associated with C–H (Aliphatic, Aromatic, and/or Aldehyde), O–H (alcoholic and/or phenolic) and N–H stretching in amino group [66,67]. Nevertheless, C–O–H bending of carboxylic group [68], and characteristic bands at 1398.88 and 1044 cm^−1^ confirming the presence of C–N and C–O stretching vibrations in aromatic compounds in the extract [50,69]. Similar functional groups were reported to be present in *Prosopis juliflora* mediated ZnO NPs [70]. However, the peaks located between 723.01 and 907.21 could probably be due to C–H stretching of alkane in the extract [71].

After the calcination of the nano ZnO samples, there were significant changes in the peak positions and absorbance as a result of structural changes in terms of particle size [72]. This changes also confirm that, the biomolecules in the plant extract acted as reducing agents in the formation of nanoparticles [73]. For instance, O–H stretching vibration mode of hydroxyl groups in the raw extract shifted from 3117.49 cm^−1^ to 3169 cm^−1^ and 3193 cm^−1^ in the nano ZnO sample (ZnO NS_60_ and ZnO NS_400_) respectively. The strong peak at 1640.63 cm^−1^ and 1630.77 cm^−1^ for the biosynthesized nano ZnO samples were ascribed to C=C stretching vibration in the aromatic ring, confirming the presence of the aromatic group (Reddy et al., 2014), but the peak seems to be a weak one in the raw extract. Again, the peaks located at 1044 cm^−1^ in the raw extract reduced to 1043 cm^−1^ in the nano ZnO samples which could be assigned to C–N stretching vibrations in the amide and aromatic rings [74]. Similar bands were identified in *C. fistula*- and *M. azedarach*-mediated ZnO NPs as well as *C. albidum*-mediated Ag NPs as reported in literature [75,76]. The band at 1398.88 cm^−1^ in the raw sample increased to 1401.13 cm^−1^ after the synthesis which could be attributed to C–N stretching of the aromatic amine group as reported by Gurunathan et al., [77]. Finally, the peaks located between 576 cm^−1^ for the synthesized nano ZnO samples could probably be assigned to the stretching of Zn–O bonds. Similar peak range was also associated with Zn–O bonds as reported by Santhoshkumar et al., [78].

### 3.3 Entrapment efficiency of ZnO nanocrystalline samples

The efficiencies corresponding to *E. radiata* mediated nano ZnO sample calcined at lower temperature (ZnO NS_60_) and higher temperature (ZnO NS_400_) were 61.16 ± 0.3% and 73.8 ± 0.6% respectively. The higher entrapment efficiency could be associated with the higher volume of extract used (70 ml) as compared to the lower entrapment efficiency using 50 ml of the extract. Interestingly, our results were found to be lower than a study by Halanayake et al. [52], that employed *Plumeria* leaf extract loaded on chitosan (65–82%). However, our study gave a higher efficiency than a study by Samling et al. [79], where *Cynometra cauliflora* essential oils loaded-chitosan nanoparticles (38–44%) was evaluated for their antioxidant, antimicrobial and cytotoxic activities.

### 3.4 Antimicrobial analysis

Table 2 shows the antimicrobial activity of the two nano ZnO samples calcined at 60 °C and 400 °C from the aqueous leaf extract of *E. radiata* against four bacteria strains and two fungal strains.

**Table 2:** Effect of *E. robusta Sm* leaf extract mediated ZnO nanoparticle on selected bacteria.

Conc. (mg/ml)	ZnO NS Type	Mean inhibition zone ± SEM (mm)						P-values	
		*E. coli* (Gram -ve)	*S. typhi* (Gram -ve)	*S. aureus* (Gram + ve)	*K. pneumonia* (Gram + ve)	*P. digitatum* (Fungi)	*C. albicans* (Fungi)	Concentration	ZnO NS Type
Control		15.3 ± 0.2	13.8 ± 0.3	21.3 ± 0.6	19.7 ± 0.2	16.7 ± 0.3	18.5 ± 0.4		
3.125	ZnO NS_60_) ZnO NS_400_	4.4 ± 0.1 5.3 ± 0.2	3.2 ± 0.1 4.6 ± 0.2	6.7 ± 0.2 8.6 ± 0.5	5.5 ± 0.4 7.9 ± 0.3	7.3 ± 0.5 8.4 ± 0.2	6.4 ± 0.4 6.9 ± 0.3	0.001	0.979
6.25	ZnO NS_60_ ZnO NS_400_	5.9 ± 0.4 6.6 ± 0.1	5.7 ± 1.4 6.1 ± 0.7	8.2 ± 0.2 9.7 ± 0.5	7.3 ± 0.2 9.2 ± 0.3	7.9 ± 0.3 8.6 ± 0.1	9.2 ± 0.2 9.8 ± 0.1	0.001	0.979
12.5	ZnO NS_60_ ZnO NS_400_	6.8 ± 0.3 7.4 ± 0.1	6.3 ± 0.4 6.6 ± 0.2	11.8 ± 0.6 12.5 ± 0.3	9.6 ± 0.1 10.1 ± 0.2	10.2 ± 0.2 10.7 ± 0.5	12.5 ± 1.1 11.3 ± 0.2	0.001	0.979
25.0	ZnO NS_60_ ZnO NS_400_	8.1 ± 0.7 8.8 ± 0.2	7.3 ± 0.8 7.9 ± 0.5	14.2 ± 0.6 15.3 ± 0.2	11.1 ± 0.3 11.8 ± 0.2	10.6 ± 0.5 11.5 ± 0.4	13.4 ± 1.7 14.9 ± 0.5	0.001	0.979
50.0	ZnO NS_60_ ZnO NS_400_	9.7 ± 1.3 9.9 ± 0.2	7.9 ± 0.2 8.3 ± 0.5	16.5 ± 1.1 17.2 ± 0.1	13.8 ± 0.8 14.2 ± 0.5	12.6 ± 0.2 13.3 ± 0.1	12.6 ± 1.3 15.7 ± 0.1	0.001	0.979

The results from Table 2 showed that, the inhibitory effect of the selected pathogens was dose-dependent on the nano ZnO sample concentration. Also, the particle size also played a significant effect on the inhibition of the microbes. Thus, the smaller the particle size, the greater the effect it had on the inhibition of the microbes. This trend was observed and reported by Stan et al. [23], where ZnO nanoparticles synthesized from aqueous extracts of *Allium sativum*, *Rosmarinus officinalis* and *Ocimum basilicum* were tested against some pathogens. ZnO nanoparticles synthesized from *A*. *sativum* has the smallest particle size and showed the most significant antimicrobial activity against *S. aureus*.

From the results in Table 2, the nanocrystalline ZnO sample calcined at higher temperature at 400 °C (ZnO NS_400_) showed the most significant antimicrobial activity against the selected bacterial and fungal strains with zone inhibition diameters in the range of 4.6–17.2 mm and 6.9–15.7 mm respectively. Out of the different bacterial and fungal strains, the same sample showed an enhanced activity against *S. aureus* (17.2 ± 0.1 mm) bacterial strain and *C. albicans* (15.7 ± 0.1 mm) fungal strain at 50 mg/ml. However, the sample calcined at a lower temperature at 60 °C (ZnO NS_60_) showed the least inhibition zone (3.2 ± 0.1) at 3.125 mg/ml on *S. typhi*. These observations could be attributed to the entrapment efficiencies of the different ZnO samples. Thus, ZnONPs_400_ sample with higher entrapment efficiency ensured a greater amount of bioactive compounds incorporated in the sample which directly influenced their therapeutic potential. Other factors that may influence this action may be the release kinetics of these bioactive molecules and their interaction with the microbial cells. A similar trend was observed in the study by Umar et al. [80], where the antibacterial activity of biosynthesized ZnO NPs was reported to be more effective on *S. aureus* than on *E. coli*.

This result affirms the assertion that, biosynthesized ZnO NPs are more effective on Gram-positive bacteria than Gram-negative based on their cell structure, metabolic activities, and degree of contact of the bacteria as reported in literature [23,81,82] Upon subjecting the obtained data to multivariate test as illustrated in Table 3, the results showed that, the mean inhibition of the pathogens based on the concentration of the ZnO used gave a statistically significant difference [F (20, 4.266) = 47.14, p = 0.001; Wilks' Λ = 0.000]. However, there was no statistical significant difference between the ZnO NS type and the mean inhibition of microorganism [F (6, 3) = 0.780 p = 0.979; Wilks’ Λ = 0.780]. This could be due to the insignificant difference between the particle size of the two samples. Previous studies suggested that, ZnONP mechanisms that usually caused antibacterial action are spelt out in different ways. For instance, ZnO NPs used as antimicrobial agents releases both positively charged (hydroxyl radicals, singlet oxygen, and Zn^2+^ ions) and negatively charged (H_2_O_2_) species that result in cell impairment leading to cell death [[83], [84], [85]]. Although, the negatively charged species are unable to penetrate the cell membrane, they usually damage the proteins (DNA) and lipids, whereas, the positively charged species penetrate directly into the cell wall (internalization) and kill the bacteria [86,87].

**Table 3:** Multivariate Tests of the concentration and ZnO NS Type.

Effect		Value	F	Hypothesis df	Error df	Significance
Concentration	Pillai's Trace	3.568	6.610	20.000	16.000	.000
	Wilks' Lambda	.000	47.141	20.000	4.266	.001
	Hotelling's Trace	.	.	20.000	.	.
	Roy's Largest Root	34105.260	27284.208^c^	5.000	4.000	.000
ZnO NS Type	Pillai's Trace	.220	.141^b^	6.000	3.000	.979
	Wilks' Lambda	.780	.141^b^	6.000	3.000	.979
	Hotelling's Trace	.282	.141^b^	6.000	3.000	.979
	Roy's Largest Root	.282	.141^b^	6.000	3.000	.979

The variation in the inhibitory effect of the different microbes by the nano ZnO samples in this study may be attributed to the morphology and their distinct mechanism of action. The smaller spherical sized samples (ZnO NS_400_) may use the internalization mechanism approach whereas the bigger sized rod-like sample (ZnO NS_60_) could interact with the cell walls through ion diffusion and free radical generation, which further penetrated the cells to destroy the cellular components (DNA, proteins, and lipids) as reported by Babayevska et al., [88].

According to Khatami et al. [89], biosynthesized ZnO NPs with a size of 2.8 nm demonstrated higher inhibitory activity against *S. aureus* and *E. coli*. Also, literature reported that, antibacterial activity of some plant mediated ZnO NPs were both size and dose-dependent where the synthesized ZnO NPs samples showed better inhibition against *E. coli* at a comparatively low concentration of 10 μg/mL [[90], [91], [92]].

## 4 Conclusions

The current study successfully synthesized nanocrystalline ZnO samples using aqueous extract of *E. radiata* leaf with two morphologies based on their different calcination temperatures. The synthesized samples were agglomerated with rod-like and spherical shaped structures with particle size of 51.04 ± 17.5 nm and 49.16 ± for 60 °C and 400 °C respectively. Spectra peaks from FTIR confirmed the presence of ZnO nanoparticles in both prepared samples. Meanwhile, the two biosynthesized nanocrystalline samples were dose and size dependant on the selected microbes. Nanocrystalline ZnO sample calcined at higher temp (ZnO NS_400_) exhibited better antibacterial and antifungal activity than the sample calcined at lower temperature (ZnO NS_60_). Out of the different bacterial and fungal strains, ZnO NS_400_ sample showed an enhanced activity against *S. aureus* (17.2 ± 0.1 mm) bacterial strain and *C. albicans* (15.7 ± 0.1 mm) fungal strain at 50 mg/ml. It is important to note that, the exact mechanisms responsible for the enhanced antimicrobial activity observed in the synthesized nano ZnO samples were not fully elucidated in this study. Future studies can delve into the mechanisms to understand the interactions between the bioactive compounds in the leaf extract and the nano ZnO structures as well as their mode of action against microorganisms since this sample showed higher antimicrobial and antifungal activity. The study therefore concluded that, the findings in this study contributed to the growing body of research on the use of *E. robusta Sm* mediated ZnONPs as a potential antimicrobial agent.

## Funding

The authors received no specific funding for this work.

## Additional information

No additional information is available for this paper.

## Data availability statement

Data included in article/supplementary material/referenced in article.

## CRediT authorship contribution statement

**Eric Kwabena Droepenu:** Writing – original draft, Methodology, Investigation, Formal analysis, Data curation, Conceptualization. **Eric Amenyogbe:** Writing – review & editing, Resources. **Mercy Adusei Boatemaa:** Writing – review & editing, Software, Resources. **Evelyn Opoku:** Writing – review & editing, Software.

## Declaration of competing interest

The authors declare the following financial interests/personal relationships which may be considered as potential competing interests: Eric Kwabena Droepenu reports equipment, drugs, or supplies was provided by University of Malaysia Sarawak Faculty of Resource Science and Technology. Eric Kwabena Droepenu reports a relationship with University of Malaysia Sarawak Faculty of Resource Science and Technology that includes: non-financial support. Eric Kwabena Droepenu has patent pending to Nil. There is no activity being involved by any of the authors which could be interpreted as a conflict of interest. If there are other authors, they declare that they have no known competing financial interests or personal relationships that could have appeared to influence the work reported in this paper.
